# Microplastic uptake and impacts on crops under realistic exposure: implications for soil–plant systems

**DOI:** 10.1007/s11356-026-37686-z

**Published:** 2026-04-01

**Authors:** Shima Ziajahromi, Chris Pratt, Nikol Slynkova, Frederic D. L. Leusch

**Affiliations:** School of Environment and Science, Australian Rivers Institute, Griffith University, Gold Coast Campus, Southport, Gold Coast, Qld 4222 Australia

**Keywords:** Crop plants, Environmentally realistic, Microplastics, Nanoplastics, Soil, Toxicity, Uptake

## Abstract

Increasing micro- and nanoplastic (MNP) contamination of agroecosystems has become a significant concern due to their potential effects on crops and food safety, yet their fate and effects under environmentally realistic conditions remain poorly understood. Here, we investigate the behaviour, toxicity and uptake of MNPs under realistic exposure conditions, considering polymer types, size, concentration and surface characteristics, in two crop plants: wheat (*Triticum aestivum*) and tomato (*Solanum lycopersicum*) in a silty loam soil, under both individual and mixed exposure. Plant presence significantly reduced the leaching of aged polyethylene terephthalate (PET) fibres and polyethylene (PE) fragments in soils, with smaller MPs (average 124 µm) leaching more than larger MPs (average 568 µm), implying a plant-mediated retention effect. Fibres caused greater effects than fragments, particularly in tomato, reducing chlorophyll content and plant growth. Notably, mixed MNP exposures induced more pronounced phytotoxicity in tomato than single exposures, suggesting potential synergistic effects. Although pristine NPs (∼400 nm) were not taken up, we found that aged NPs can be absorbed by plant and translocated to both roots and stem base tissue in both species, with further translocation to the leaf vascular system observed in tomato, highlighting the impact of surface characteristics on the uptake. This study provides new evidence on the effects and uptake of environmentally representative MNPs, addressing a critical gap in current research that mostly relies on pristine MNPs and at unrealistic concentrations. Our findings highlight the urgent need for risk assessments and regulatory frameworks that reflect real-world conditions to protect sustainable agriculture and public health.

**Supplementary Information:**

The online version contains supplementary material available at 10.1007/s11356-026-37686-z.

## Introduction

Microplastics (MPs; plastic particles less than 5 mm in size) and nanoplastics (NPs; plastic particles less than 1 µm in size) are expected to accumulate in agroecosystems at concerning rates due to their continual input from various sources such as biosolids, composts, plastic mulching, mismanaged plastic used in farming practices (e.g. crop covering), surface run-off, and even atmospheric deposition (Okeke et al. 2022; Zhou et al. 2021). Micro- and nanoplastic (MNP) contamination of agricultural soils is irreversible and can threaten entire ecosystems (Yadav et al. 2022).

The fate of MNPs within the soil–plant system, potential uptake by crop plants and the subsequent implications for plant health and food safety are areas of great concern (Chae and An 2018). Recent studies suggest that MNPs can alter soil properties, impact soil microbial communities, change soil enzyme activity and disrupt the nitrogen and carbon cycle in soil (Okeke et al. 2022; Zhou et al. 2021). This raises significant concerns regarding the toxicity of MNPs to plants and their broader ecological and human health implications. Research on terrestrial plants has indicated that MNPs could inhibit seed germination, adhere to root surfaces, inhibit photosynthesis, trigger oxidative stress and cause cellular and genetic toxicity (Colzi et al. 2022; Ren et al. 2024; Zhou et al. 2023). The uptake of MNPs in plants has received attention over the past few years, with studies demonstrating the translocation of MNPs from root to shoot and leaves (Azeem et al. 2021). To reach the vasculature and be transported to shoot, MNPs must cross several physiological barriers, including the cuticle, epidermis, cortex, endodermis and the Casparian strip (Yang et al. 2023). Therefore, particle size is a key factor limiting their uptake and transport into plants. Recent studies have shown that submicron NPs (200 nm) could be taken up by the root apex of crop plants such as wheat and lettuce (Huang et al. 2022). It is expected that as particle size decreases, the number of MPs increases, leading to higher environmental concentrations of small-sized and more harmful plastics (Leusch et al. 2023). Yet, the impact of MNPs on terrestrial plants under environmentally relevant conditions (e.g. aged vs virgin plastics, realistic concentrations, sizes, polymer types and morphotypes) is not well understood, limiting our ability to assess their actual environmental risks.

Understanding the toxicity and uptake of environmental MNPs in crop plants is crucial for assessing their risks to both human and ecosystem health (Liu et al. 2023). With MNPs now detected in various organs and tissues of the human body including the liver, lungs, blood, placenta, testicle and even the brain (Amato-Lourenco et al. 2024; Hu et al. 2024; Ragusa et al. 2021), it has become urgent to identify and mitigate human exposure to MNPs. Dietary exposure through crop foods is an important pathway for MNPs to enter the human body (Li et al. 2024). The presence of smaller MNPs (< 50 µm) has already been documented in some crop plants such as apple, corn, carrots, cucumbers, lettuce, onion, maize, wheat, rice and barley (Oliveri Conti et al. 2020; Yang et al. 2023). However, our knowledge of MNPs uptake from contaminated soil, and their impact on crop growth and health, remains limited. Indeed, many studies that have investigated the terrestrial fate of MNPs lack consistency in experimental design, which affects the interpretation, applicability, and comparability of findings for effective risk assessment. One major barrier to assessing true MNP risk is the use of unrealistic concentrations and characteristics that do not accurately represent soil conditions. This approach fails to capture real-world scenarios and limits the policy applicability of research findings. For example, a previous study by Li et al. (2020) showed the uptake of submicron MPs (200 nm) by wheat plants, which were largely based on using pristine NPs at concentrations several orders of magnitude higher than those typically found in agricultural soils. While valuable for the mechanism insight, this does not reflect real-world scenarios and thus limits policy applicability. Previous research showed that aged MPs have greater toxicity and increased bioavailability to exposed organisms (earthworms) than their pristine counterparts (Jiang et al. 2023). In addition, although MNPs in the soil environment typically occur together, previous studies have primarily focused on the impact of individual MNPs on terrestrial organisms, thereby failing to capture the real-world complexity of MNP exposure. Consequently, our understanding of the impact of MNP mixtures remains largely limited. In a recent study, Seo et al. (2025) highlighted the importance of research investigating the impacts of mixed MPs to better simulate actual soil MP contamination scenarios. 

To address these critical knowledge gaps, this study aims to investigate the fate, toxicity, and uptake of MNPs in two major crop plants (wheat and tomato) under environmentally realistic exposure conditions, taking into account concentrations, size, surface characteristics, polymer types, and shapes commonly found in biosolids—a major source of MPs in agricultural soils—and in agricultural soils themselves, using both single and mixture exposure. Furthermore, we examined for the first time the influence of plants on the vertical mobility and retention of environmentally representative MPs in soil. This study not only encapsulates ecological realism; it also provides critical, policy-relevant data needed to guide sustainable land management and risk assessment frameworks in the face of increasing plastic pollution in agricultural soils.

## Materials and methods

### Microplastics preparation and characteristics

This study used two types and two shapes of MPs predominantly found in biosolids and agricultural soils including polyethylene (PE) fragments and polyethylene terephthalate (PET) fibres within the size range of 25–500 µm (Ziajahromi et al. 2024). We also used submicron (∼400 nm) polychromatic red polystyrene (PS) NPs with excitation wavelength 520 nm (Polyscience, USA) to monitor potential uptake of smaller MNPs by crop plants, as this excitation makes the NPs easily distinguishable from the natural fluorescence of plant tissue. A recent study identified PS NPs in agricultural soils in France (Wahl et al. 2021), although it could not quantify their concentration due to a technical limitation.

For PET and PE MPs, we used two environmentally relevant MP concentrations including a high concentration reflecting the levels of MPs found in biosolids (a major source of MPs in agricultural soil) and a low concentration representing levels reported in agricultural soils. Specifically, low concentrations ranged from 0.2 to 0.6 μg/g, and high concentrations ranged from 900 to 9600 μg/g. Due to limited environmental data on NPs in soils, PS NPs were applied at low and high concentrations based on reported surface water levels, ranging from 0.002 to 0.05 μg/g. A mixture of PET, PE and PS was applied at corresponding low and high concentrations within this range. Detailed concentrations and characteristics of all MNPs used are provided in Table 1.

**Table 1:** Concentration and characteristics of MPs/NPs used for the experiments

Morphotype	Polymer type	Size range (μm)	Concentration (low and high)	Reference
Fragment	Polyethylene (PE)	25–500	L, 0.2 μg/g H, 9600 μg/g	Zhang et al. (2020) Okoffo et al. (2020)
Fibres	Polyethylene terephthalate (PET)	25–500	L^*^, 0.6 μg/g H, 900 μg/g	Wang et al. (2021) Okoffo et al. (2020)
Bead	Polystyrene (PS)	0.4	L^**^, 0.002 μg/g H, 0.05 μg/g	Xu et al. (2022) Sun et al. (2020)
Mixed^***^	30% (PET), 60% (PE) and 10% (PS)	0.4–500	L, 0.18 μg/g (PET), 0.12 μg/g (PE), 0.0002 μg/g (PS) H, 0.27 μg/g, (PET), 5760 μg/g (PE), 0.005 μg/g (PS)	

^*^1.35 particles/g: the conversion to mass was performed using the median size, polymer density and based on the formula proposed by Leusch and Ziajahromi (2021)

^**^Based on the environmental concentration found in surface water samples

^***^Calculated based on the concentration of individual MPs

MP fibres were prepared using a fleece clothing item as described in our previous study (Ziajahromi et al. 2017), and wet sieved using a filtration device consisting of stainless steel filters including 25 and 500 µm to separate the desired size range. The separated particles were soaked in methanol (Sigma-Aldrich, 99.9%) to remove potential contamination and then washed three times with ultrapure water and filtered through a 25 µm filter using vacuum filtration. The size of the produced fibres was confirmed by laser diffraction (Malvern Mastersizer 3000) to ensure that the specific size range was achieved for each plastics type and shape. Information about the size range analysis of fibres is provided in Section S1 Table S1 in the Supporting Information.

MP fragments were prepared using a multi-step processing technique developed in-house to generate micro-fragments. A coffee grinder was used to create larger plastic fragments from an unused PE take-away container. The created fragments were transferred to 10 mL stainless steel grinding jars (Qiagen, USA), and three small stainless steel grinding balls were added to the jars. The jars were left in liquid nitrogen for 10 min and then vigorously mixed for 2 min using a Tissue Homogenizer (TissueLizer II, QIAGEN, Germany). This step was repeated three times. The created MPs were wet-sieved using a filtration device already mentioned above. MPs between 25 and 500 µm were separated. The prepared MPs were soaked in methanol (until all methanol evaporated) to remove potential contamination, washed with ultrapure water, and their size range was determined as described above.

To better simulate environmentally relevant MNPs, all MNPs were aged in a custom-built ageing chamber (Figure S2), designed and built at Griffith University following the method by Wang et al. (2020). This was performed based on a preliminary assessment of the oxidation level of MPs extracted from agricultural soils in our previous study (Ziajahromi). The detail of the ageing process is provided in Section S2 (and Figure S1). The aged MPs were characterised using FTIR (Thermo Fisher, USA), and their zeta potential was obtained using Zetasizer (Malvern Nano-SZ) to assess their oxidation state and surface charge. The carbonyl index, a widely used metric for assessing the ageing of MNPs (Rouillon et al. 2016), was calculated from FTIR spectra of aged and referenced MNPs by dividing the absorbance at the carbonyl peak (1710–1740 cm⁻^1^) by the absorbance at a stable reference peak (PE 1460 cm⁻^1^, PET 1409 cm⁻^1^, PS 1452 cm⁻^1^) (Prata et al. 2022).

To enable tracking of targeted MNPs during exposure, MPs were stained using a non-toxic fluorescent dye (i-dye pink; Jacquard) according to Karakolis et al. (2019) methodology.

### Experimental set-up

Seeds of tomato (*Solanum lycopersicum*) and wheat (*Triticum aestivum*) were used in this study. These crop plants were selected because of their fast life cycle and because wheat is the largest grain crop produced in Australia, while tomato is among the most widely consumed vegetables in the country. A silty loam agricultural soil (classified as Planosol; Table S2) was collected from an uncontaminated site with no history of biosolid application, mulching or any other plastic input. The soil was sieved to 2 mm and dried in an oven (at 50° C) for 24 h. Background MP contamination of the soil was measured by analysing sub-samples of the collected soils. Three sub-samples (100 g) of soil were processed according to our previous method, and suspected MPs were analysed using FTIR spectroscopy (Ziajahromi et al. 2024).

MPs at desired concentrations were added to 25 mL glass vials with 20 mL ultrapure water. Tween 20 (1% v/v) was added to disperse MPs in water following our previously published method (Ziajahromi et al. 2017), and the mixture was vortexed for 2 min. Soils (500 g) were transferred to glass jars, and prepared MNPs were spiked into the soil at the nominal concentrations. The soil was then dried at 50 °C and thoroughly mixed with a glass spatula to ensure distribution of MNPs within the soil. The soil was then transferred to the experimental pots. MP-spiked soils at each concentration level were tested to confirm the actual concentration of MPs in soil. A 50 g subsample of the soil was processed following our previously published method, and MPs (25–500 µm) were counted using a fluorescent stereo microscope (SZX10, Olympus) through a semi-automated approach using Cellsense image analysis software (Ziajahromi et al. 2024). The counted MPs were then converted to mass based on the approach described by Leusch and Ziajahromi (2021) to match the results with the initial concentration used in the experiments. The details of the soil sample processing method and information of actual concentrations of MPs are provided in Table S3. The actual concentration of MPs was within 80–120% of nominal concentrations which are within the acceptable level of ± 15–20% outlined by the OECD guideline for toxicity testing of chemicals (OECD 2018). Due to technical limitation in quantification of nano-sized plastics using stereo microscopy, the actual concentration of PS NPs in soil could not be confirmed.

To examine the impact of plants on the movement of MNPs within the soil, two control setups were included in each experiment: one set of control pots containing MNPs without plants and one set of control pots with plants but without MNPs. Each treatment was conducted with four replicates. The treatments for each crop plant included the following: PET fibres, PE fragments, PS NP beads, and one mixed treatment (30% PET, 60% PE and 10% PS NPs) based on previous studies reporting MPs in agricultural soil (Piehl et al. 2018; Sharmin et al. 2024; Xu et al. 2022), each at two environmental concentrations (low and high) as specified in Table 1. To further investigate uptake of NPs, an additional treatment was included using pristine NPs with four replicates. These treatments aimed specifically to assess if particle ageing influences plant uptake of NPs. In total, 120 experimental pots were set up.

The temperature in the facility was set to 22 °C with a constant relative humidity of 60%. Fertilizers containing nitrogen, phosphorus and potassium were added to each pot (Table S4). A full-spectrum artificial light (LED plant-tube full spectrum—Lytworx) was used as the light source above each rhizotron (16 h light/8 h dark). All plants were exposed to MNPs for the duration of their growth cycle (3–5 months), until the control pots (without MNPs) reached maturity. Upon the completion of the experiment, the entire plants (including shoots and roots) were removed from the pots and placed on aluminium foil for microscopy and the assessment of toxicity endpoints (Fig. S2).

### Leachate collection and analysis of MNPs in leachate

To examine the fate and movement of MNPs between soil and water, leachate was collected regularly during the experiment period using a pour-through method (Altland and Owen 2024) with some modifications. Briefly, this included watering the pots thoroughly with ultrapure water until water started to drain from the bottom (saturate the substrate), ensuring the substrate was at its water-holding capacity. The leachate was collected immediately as water first appeared from the pot and continued for 30–60 min to allow full drainage. The leachate from each pot was collected in a pre-cleaned (500 mL) glass jar. The leachate samples were processed in the laboratory according to previous methods (except for the digestion step) (Sheriff et al. 2024; Vural et al. 2025). The processed samples were filtered through 25 μm stainless steel filters and dried in an oven at 40 °C. The dried filters were inspected under a fluorescent stereo microscope (SZX10, Olympus) equipped with a digital camera. Particle counting and sizing were performed through a semi-automated approach using image analysis microscopy software (cellSens imaging software, Olympus). Due to logistical issues in counting PS NPs using a stereo microscope (optical resolution ~ 0.8 µm, with practical size detection typically in the 5–20 µm range), this step was only performed for PE and PET treatments.

### Phytotoxicity assessment

All plants were inspected under a fluorescent stereo microscope to assess the attachment of MNPs to their external surface, particularly around the shoots and roots. They were then washed with ultrapure water to remove any residual soil and surface MNPs. After washing, the plants were re-inspected under the stereo microscope to detect the presence of absorbed MNPs in the roots, shoots and leaves.

Phytotoxicity endpoints were measured to assess the impact of MNPs on plants (Schmidt and Redshaw 2015). These endpoints include visual assessment of plant viability according to Hu et al. (2022). Plant growth was assessed by measuring parameters such as shoot length, root length, organ emergence, root biomass and chlorophyll content as an indicator of photosynthesis performance (Song et al. 2023). Total root length was measured using a line transect method (Pandey et al. 2017). In this method, grid paper (1 cm × 1 cm) was placed beneath a glass sheet. The roots were then carefully spread over the moistened glass surface. The total number of intersections between the grid lines and the root is counted. The total root length is then calculated using the following equation (Eq. 1):

1 $$\mathrm{Total}\;\mathrm{root}\;\mathrm{length}=0.786\times\mathrm{no}.\;\mathrm{of}\;\mathrm{intersections}$$

Root biomass was measured using the gravimetrical method by recording both fresh weight and dry weight (dried in an oven at 65 °C for 72 h) of root samples (Füzy et al. 2019).

Photosynthesis was assessed by measuring the total chlorophyll content. Leaves were cut into small pieces using scissors, and 0.5–1 g of leaves were transferred to 100 mL beakers. Acetone (Sigma Aldrich, 99.9%) was added until the leaves were fully submerged. The beakers were placed on a shaker for 5 min to ensure thorough extraction. The extract was transferred to centrifuge tubes and centrifuged for 5 min at 1000 g. Absorbance reading was performed using a UV spectrophotometer (UV-1800 Shimadzu, Japan) at wavelengths of 663 nm and 645 nm. Chlorophyll a, b and total were measured according to the formula described in Perez-Patricio et al. (2018).

### Quality assurance/quality control (QA/QC)

To ensure data integrity and quality, a strict QA/QC protocol, consisting of 20 quality criteria in four main categories; particle characterisation, experimental design, applicability for risk assessment and ecological relevance was followed throughout this study as recommended by de Ruijter et al. (2020). This included verification of soil background contamination and confirmation of the nominal MP concentration. Additionally, to support ecological relevance and applicability for risk assessment, our study ensured the use of environmentally realistic MNP concentration, type, form and surface characteristics. To minimise background contamination, non-plastic equipment and containers were used whenever possible. All containers were pre-cleaned with ethanol and ultra-pure water, and cotton lab coats were worn.

### SEM imaging

The regions of stem, root and leaf identified as containing NPs through fluorescent microscopy were selected for further analysis using Scanning Electron Microscopy (SEM). The selected samples were sectioned into small pieces and freeze-dried for 48 h (Virtis Genesi, USA). They were then vacuum-dehydrated and coated with 2 nm thickness of gold to minimise charging issues. Cross sections were examined using SEM (MIRA3, TESCAN) at an accelerating voltage of 5 kV. The images were captured at different magnifications.

### Statistical analysis

Phototoxicity data acquired were analysed and visualised using GraphPad Prism (v9.3.1). Normality of data was assessed using the Shapiro-Wilk test. Following this, a parametric one-way ANOVA was used to examine the statistical significance of the different treatment groups with control, with a significance level set at *α* = 0.05. Two-way ANOVA was used to analyse how the shape and type of MPs influence the vertical movement of MPs in soil for soil leachate experiments. Where significant differences were observed, a post hoc Tukey’s multi-comparison test was performed to compare multiple treatment groups. All statistical analyses were performed with *α* = 0.05.

## Results and discussion

### Characteristics of MNPs

Aged MNPs showed an increase in carbonyl index compared to pristine MNPs associated with oxidative degradation and formation of oxygen-containing functional groups on the MNP surface during the ageing process. Both aged and pristine MNPs had negative zeta potential with aged MNPs consistently showing more negative charge after ageing (Figure S3). This reduction in surface charge is likely attributed to the formation of polar carbonyl groups, which can alter surface chemistry and reduce electrostatic repulsion. This change may influence aggregation behaviour of MNPs and enhance their mobility in the system. For example, Liu et al. (2019) reported that UV-aged PS NPs showed increased mobility in loamy sand.

The size range of MNPs is provided in Table S1. The PET fibres ranged from 37.8 to 824 µm, while PE fragments had a size range of 28.1 to 552 µm. The larger fibre size can be attributed to their fibrous morphology (thin and elongated structure) which allows the larger fibres to pass through smaller filters.

### MPs in leachate samples

**Fig. 1:**
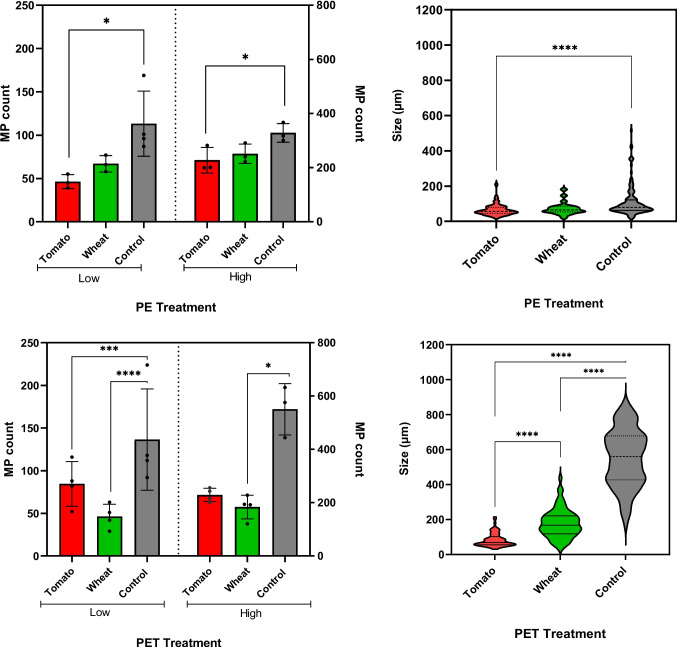
Size and abundance of MPs (≥ 25 μm) detected via fluorescent stereo microscopy in the soil leachate samples. Control treatments are those without plants. Data are reported as average ± SD. Asterisks indicate levels of statistical significance as follows:; ****p < 0.0001. ***p = 0.001; **p = 0.01; *p = 0.05. Note: High and Low refer to the concentrations of MPs for each treatment

**Fig. 2:**
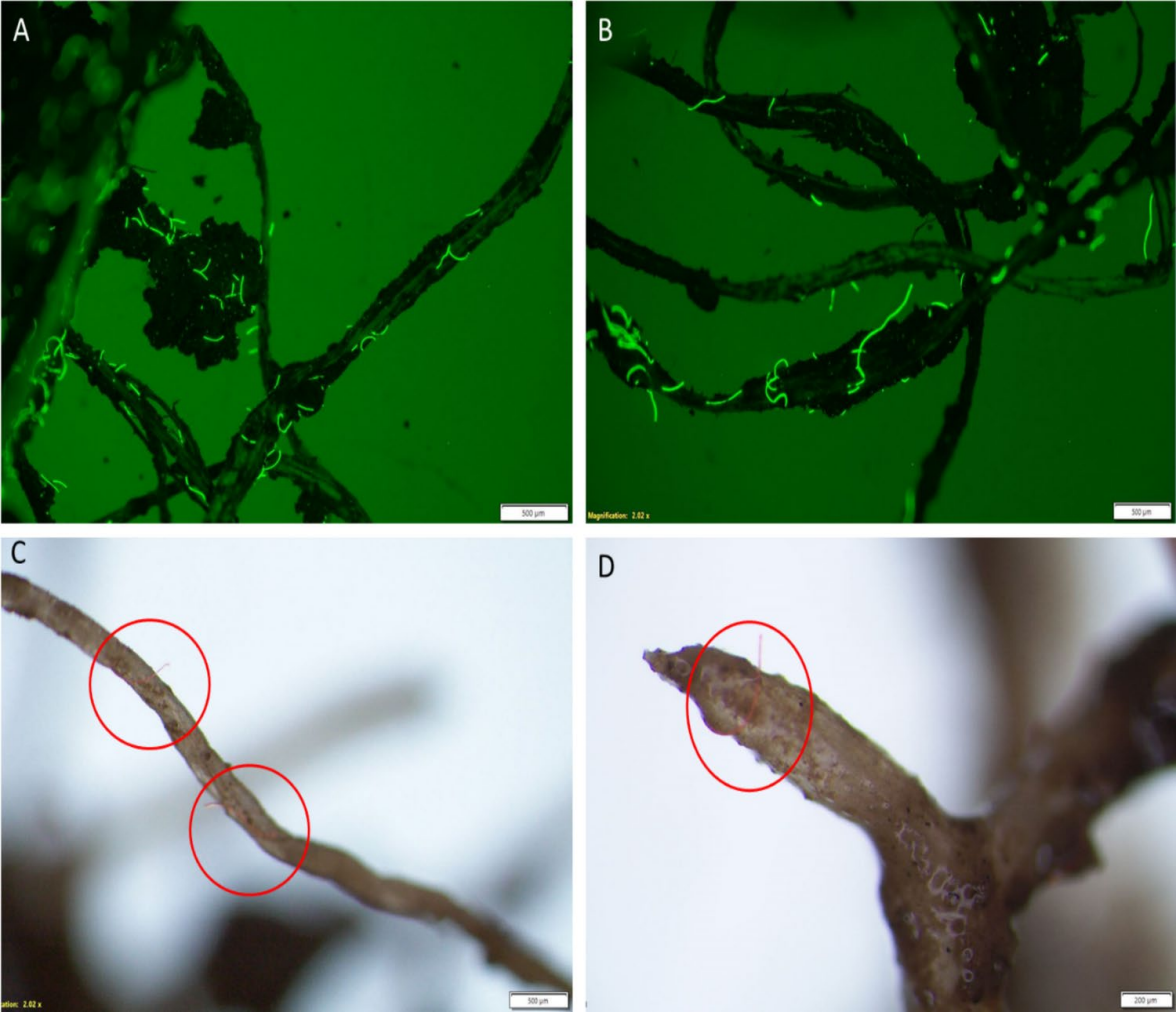
Microscopy images of PET fibres on the external surface of the plant roots. **A**, **B** PET fibres around root hairs and rhizospheric soil of wheat plant. **C**, **D** PET fibres physically attached to root tip and hair of tomato plant

To understand the behaviour and movement of MPs within soil, it is important to monitor their transport from soil to water leachate in the presence and absence of plants as the plant root may influence the movement and retention of MPs within the soil profile. Figure 1 shows the abundance and size of MPs detected in leachate samples. The results for PET fibres in both tomato and wheat treatments showed significantly (*p* < 0.001) smaller average size range of MPs (124 ± 82.2 µm) in the leachate compared to the control group without plants (568.6 ± 165.4 µm). One possible explanation is that larger fibres were retained within the rhizosphere, potentially due to entanglement or physical attachment to root hair, which may have limited their mobility through the soil matrix. In addition, smaller MPs are more mobile and can leach more readily from soil than larger particles, with the silty loam soil in this study likely contributing to their mobility by allowing easier movement through its interconnected pore spaces. Our microscopy data clearly showed the presence of larger fibres surrounding and attached to the root surface in both plant types, highlighting the role of plant roots in entrapping larger MPs (Fig. 2 and Figure S4). Therefore, both plant root systems and soil properties contribute to the retention and accumulation of MPs in soil, particularly for larger particles. Wheat, a monocot, has a fibrous root system with highly branched roots with larger surface area but relatively loose packing, which may facilitate movement of smaller PE fragments through the rhizosphere and into leachate (Tumwet et al. 2024). In contrast, tomato, a dicot, has a taproot system with dominant central root and fewer lateral branches, which may more effectively trap elongated PET fibres, ultimately reducing their mobility into leachate. In a recent study, Tumwet et al. (2024) showed that wheat’s fibrous root system influenced the vertical movement of MPs, with polyester fibres showing strong attachment to root while PVC fragments showed greater mobility through the soil profile. This could also highlight the potential of plants for in situ remediation of soil contaminated with MPs (Yuan et al. 2024).

Likewise, a higher number of PE fragments (*p* < 0.001) was observed in the leachate of pots without plants (control samples) compared to the pots with plants (Fig. 1). The presence of plants appears to reduce MP leaching by enhancing soil structure and promoting the MP retention through root interactions (Yuan et al. 2024). This interaction was more significant for PET fibres, with more fibres retained in the soil by the plants compared to PE fragments. The results showed that, on average, between 42 and 65% of all PET MPs in the soil leached in the absence of plants, whereas only 14% to 26% leached in the presence of plants. For PE fragments, on average, 23 to 48% leached in the absence of plants, while only 13% to 21% leached when plants were present. Apart from the influence of plants in the movement of MPs in soil, the shape of the MP itself could play an important role in vertical infiltration. MP fibres are elongated and usually have a smaller diameter than fragments which can facilitate their movement through the soil pores. A previous study by Qiu et al. (2023) found fibres in all soil layers, whereas fragments were only found in topsoil layers, indicating that MP fibres may have higher vertical mobility than fragments. This highlights the potential for fibres to migrate into deeper soil layers, potentially reach groundwater and disperse throughout wider ecosystems. Previous laboratory studies using pristine (non-aged) MPs showed that higher density MPs (like PET) have higher gravitational sedimentation, and thus increased retention in soil than lower density MP fragments (like PE, PP) (Dong et al. 2021). Our results are in contrast with this finding, which could be attributed to the change in MP surface chemistry due to ageing, specifically an increase in surface functional groups that can reduce the deposition of MP in soil by hydration forces. Yan et al. (2020) showed ageing as a dominant factor influencing MP mobility, facilitating their downward movement in soil. This highlights the importance of using environmentally relevant MNP characteristics in fate and ecotoxicology studies as surface modification of aged MNPs affects their environmental behaviour and toxicity (Ding et al. 2024). It should be noted that MPs in soil may have undergone further fragmentation and ageing through physical abrasion, microbial degradation and chemical oxidation, although the degradation rate might be slow and polymer specific (Zhong et al. 2025). Interaction between MNPs and soil minerals and organic matter can influence aggregation, surface charge and mobility (Gong et al. 2024). Further research is required to better understand MNP degradation mechanisms (such as thermodegradation and photodegradation) in soil systems and their impact on particle reactivity, transport behaviour and bioavailability.

### Phytotoxicity assessment

#### Plant growth

**Fig. 3:**
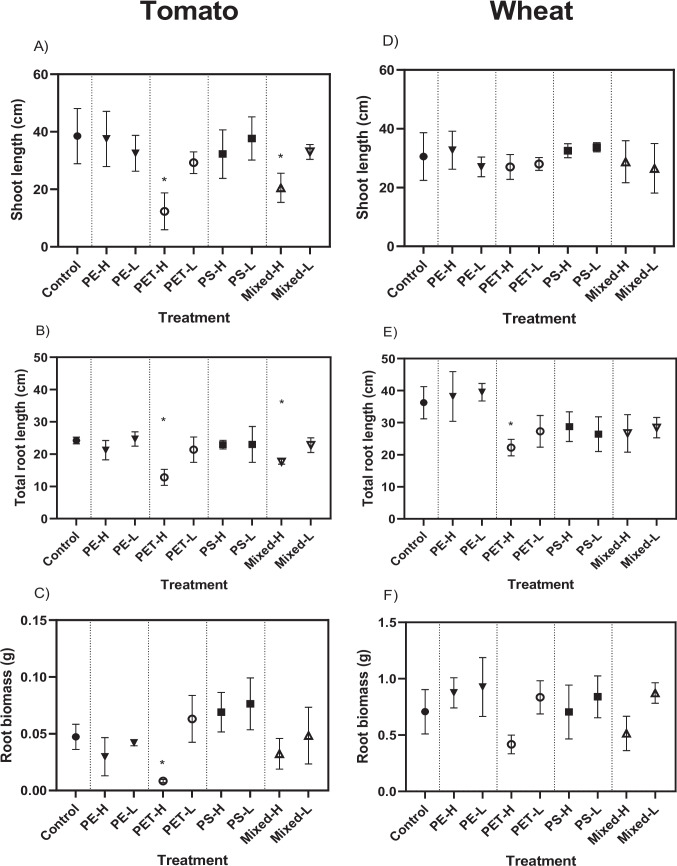
Shoot length, total root length and root biomass of tomato  (A,B,C) and wheat plants (D,E,F) exposed to MNPs at low (L; similar to soil) and high (H; similar to biosolids) concentrations. Data are reported as average±SD. Asterisks indicate statistically significant difference compared to control (p < 0.05). Control treatments refer to the treatment with plants without MNPs

The root and shoot length, as well as root biomass, are important measures of plant growth. Our results demonstrated that exposure to PET fibres at higher environmental concentrations (i.e. those found in biosolids) reduced plant growth in both species with tomato plants being more sensitive (Fig. 3). In the tomato plant, higher environmental concentration of PET fibres significantly decreased shoot length (67%; *p* = 0.004), total root length (47%; *p* < 0.0001) and root biomass (82%; *p* = 0.03) compared to the control treatment without MNPs (Fig. 3A–C, Figure S5). In wheat, while the impact of PET fibres on growth was less pronounced, the total root length was significantly reduced by 39% (*p* = 0.008) under the same PET concentration (Fig. 3E). Reductions in shoot length (11%) and root biomass (31%) were observed but were not statistically significant (Fig. 3D, F).

Exposure to higher environmental concentrations of PE and PS did not result in statistically significant changes in any of the growth parameters in either species, suggesting less toxic impact of fragments and beads than fibres. However, exposure to the mixed MNPs induced more adverse effects on growth of both species. In tomato, the co-exposure of MNPs significantly decreased both shoot length (47%; *p* = 0.008), and total root length (27%; *p* = 0.018), indicating mixture effects of different MNPs (Fig. 3A, B). Mixture exposure induced greater effects than predicted from single exposures alone, suggesting potential additive or synergistic interactions. In wheat, the mixed treatment resulted in a reduction in total root length (26%), root biomass (27%) and shoot length (7.6%), although none of these changes were statistically significant (Fig. 3D–F). This finding emphasises the ecological relevance of studying mixtures of MNPs to effectively estimate their true environmental impact and risks.

Although plant roots did not appear to absorb large MPs, more adverse impact of fibres on plant growth may be explained by physical interaction of fibres with the roots and accumulation in the rhizosphere. In this study, greater attachment of fibres to root was observed compared to fragments, which may have caused the stronger growth inhibition. Due to their elongated morphology, higher aspect ratio and larger size (up to 800 µm; Table S1), fibres are more likely to entangle within the rhizosphere and adhere to root surface potentially restricting root elongation and interfering with nutrient and water uptake (Jia et al. 2023). Recent studies have shown that larger MPs also alter soil structure, reduce pore connectivity, and influence microbial communities and nutrient cycling processes (Liu et al. 2026). Zhou et al. (2023) showed that the impact of polyester (PES) and PP MPs on crop plants is complex and dependent on the type of plant species. For example, PES only reduced total biomass of soybean (*Glycine max*), while PP MPs reduced total biomass of corn (*Zea mays*), peanuts (*Arachis hypogaea)* and soybean.

Zeb et al. (2022) investigated the impact of large pristine PES fibres (average length of 2.5 mm) on lettuce plants and showed that PES significantly reduced growth, chlorophyll content and antioxidant enzyme activity. Their study also showed a change in the metabolite profile of lettuce leaves as well as a change in the structure of rhizospheric bacteria with some changes reported to key beneficial bacteria involved in the carbon and nitrogen cycles.

These findings raise concerns, as fibres are the dominant form of MPs entering the agricultural environment through various pathways (e.g. biosolids, compost, atmosphere deposition), and they have the ability to infiltrate deeper into the soil and interact with the crop root system, consequently affecting overall plant health.

Previous studies have shown that PS MNP beads significantly reduced plant biomass and height (Xu et al. 2024) by reducing antioxidant enzyme activity and inhibiting plant root growth. However, these effects were typically observed at very high concentrations (e.g. 50 and 100 mg/kg), unlikely to occur in actual soil environments.

#### Chlorophyll content

**Fig. 4:**
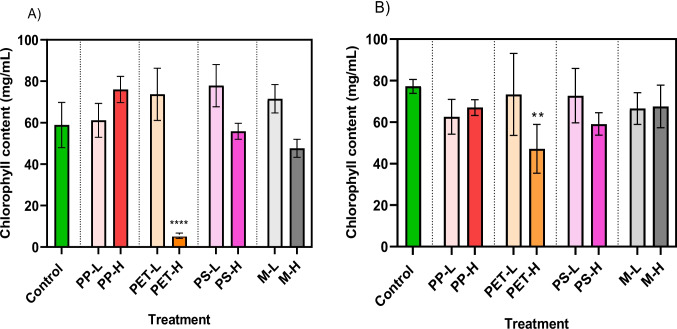
Total chlorophyll content of **A** tomato and **B** wheat plants exposed to MNPs at low (L; similar to soil) and high (H; similar to biosolids) concentrations. Asterisks indicate statistical significant differences compared to control treatments; ****p < 0.0001; **p = *0.006*

As expected, a significant decrease in chlorophyll content was observed at high environmental concentration of PET fibres in both tomato and wheat plants (*p* < 0.001 and *p* = 0.006, respectively) (Fig. 4). The decrease in the chlorophyll content is directly associated with the significantly lower tomato plant growth observed in the PET treatment compared to the control. A reduction in chlorophyll content was observed at high PS concentration in both plants, although these changes were not statistically significant. Mixed exposure at high concentration caused reduction of chlorophyll content by 25% and 12% in tomato and wheat plants, respectively. Previous studies showed that pristine PET fibres significantly affected chlorophyll content and photosynthesis in rice (at 5 mg/kg) and capsicum (at 200 mg/kg) (Yang et al. 2025). In another study, Zhou et al. (2023) reported that the effects of PP and PES MP fragments on the photosynthetic efficiency of three crop plants (corn, soybean, and peanuts) were limited. This study concluded that the impact of MPs on plants is species-specific.

### Uptake and accumulation of MNPs in plant tissue

**Fig. 5:**
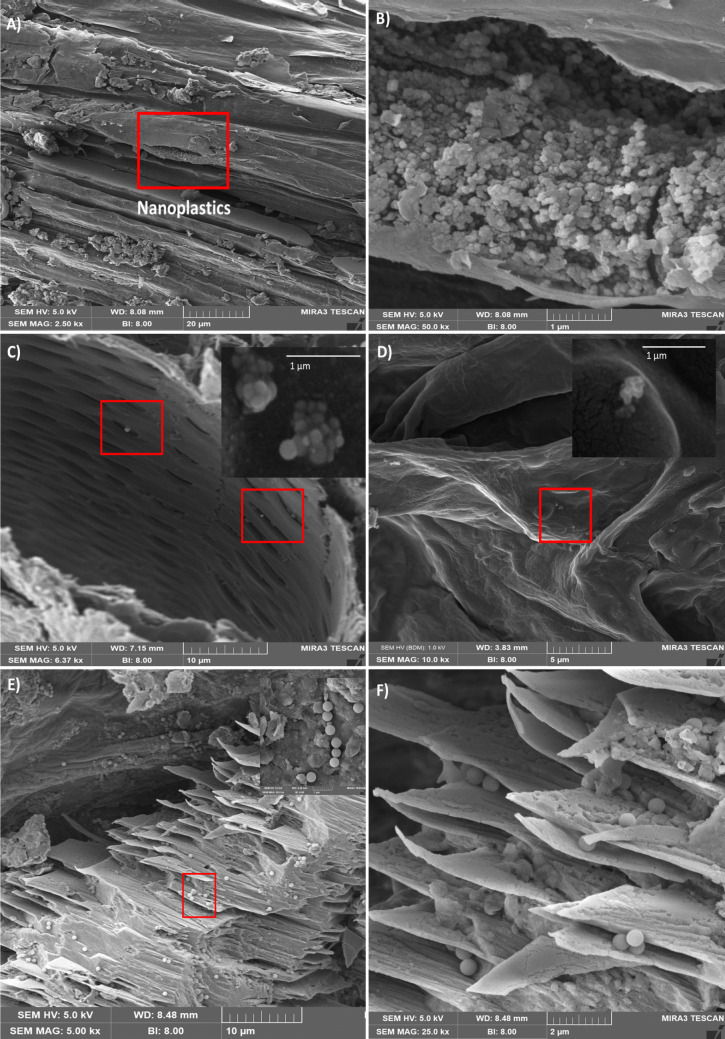
Scanning electron microscopy (SEM) images of plants exposed to 0.05 μg/g PS NPs. **A**, **B** Chain-like shape cluster of PS NPs in wheat stem base; **C**, **D** small grape-shape cluster of PS-NPs observed in tomato leaf vascular tissue; **E**, **F** PS-NPs observed in the vascular tissue of tomato roots (appearing as a layered structure likely caused by breakage during sample preparation and dehydration for SEM imaging)

While the impact of PS NPs on plant growth parameters and chlorophyll content was negligible in both crop species, our results showed that aged NPs at 0.05 μg/g were taken up by the plant root and were observed in both the root and lower stem region of tomato and wheat plants (Figs. 5 and Figures S6, S7, S8). In tomato, aged NPs were also observed in the leaf (Fig. 5C and D, Figure S7). In wheat plants, aged NPs were only observed in a confined region of the stem base, showing higher local accumulation compared to tomato plants, where aged NPs were more widely distributed but accumulated to a lesser extent. It should be mentioned that detection of NPs was performed qualitatively using fluorescence microscopy and SEM, and quantitative particle enumeration (e.g. particles g⁻^1^ tissue) was not conducted due to current methodological limitations associated with reliable extraction and quantification of sub-micron particles in this matrix.

While it is generally expected that only very small (< 50 nm) nanoparticles can be taken up by plant roots through the known pathways such as circular pores, stomata and crack-entry, previous studies have reported the uptake of submicron nanoparticles of > 230 nm by plant roots (Azim et al. 2022). One potential mechanism for the entry of submicron MPs into root tissue is through apoplastic and symplastic pathways, whereby NPs can penetrate root epidermal cells and move across cell walls or through plasmodesmata, eventually reaching the vascular system and being translocated to aerial parts of the plant (Zhang and Su 2024). Following root entry, particles may be transported upward through the xylem along with the transpiration stream, driven by transpiration pull and stomata opening, which creates a negative pressure gradient that facilitates the upward transport of dissolved and particulate matter (Ren et al. 2024; Zhang and Su 2024). The hydrophobic nature of many polymers may also increase adherence to vascular tissue, potentially influencing their retention and localization within plant tissue (Ren et al. 2024). It is also likely that NPs entered the plants during their early growth stage, when root tips and young tissues are more permeable due to the incomplete development of the Casparian strip. Further research is required to investigate the mechanisms of uptake for environmentally relevant submicron NPs during different stages of plant growth and their translocation to different parts of the plants. In this study, we did not harvest sufficient tomato fruits from mature plants prior to the completion of this study to reliably estimate further translocation of NPs to the edible parts.

Li et al. (2020) showed that pristine PS NPs (200 nm) at a very high concentration of 50 mg/L (1000 times higher than the highest concentration used in our study) were translocated to wheat roots and were visible in the cracks between the epidermal tissue of the main root and secondary root. Similarly, our study revealed a small cluster of aged NPs located in gaps within the vascular tissue in tomato and wheat plants (Fig. 5C, D, E, F). Both fluorescent microscopy and SEM detected aged NPs in the lower stem region of wheat, which suggests a localised accumulation of NPs within vascular tissue (Fig. 5A, B). This indicates that aged NPs may accumulate preferentially in specific areas of the stem, potentially influenced by transport dynamics or structural features of the vascular system. We also observed aged NPs in the vascular tissue of tomato leaves. Importantly, and in contrast to Li et al. (2020), we did not observe any uptake of pristine NPs in either wheat or tomato plant. This difference may be attributed to the much lower concentrations used in our study, as well as the use of aged NPs with altered surface characteristics. Aged NPs often exhibit increased negative surface charge, which may enhance their interaction with plant tissues. While the mechanism of uptake of NPs is not fully understood and may vary across plant species, one important factor in uptake of aged NPs by plant roots is their surface charge. A previous study suggested that NPs with negatively charged surfaces are more likely to be absorbed by plant root hair in the maturation region and internalised into stela (Jadhav and Medynska-Juraszek 2024). Sun et al. (2020) showed that while both negatively and positively charged PS NPs (200 nm) impaired the growth of Thale Cress plants (*Arabidopsis thaliana*), negatively charged NPs were taken up more by plant roots and were observed frequently in the apoplast and xylem.

Given these findings, it is unclear whether the presence of aged NPs in vascular tissue resulted from direct-mode entry as reported by Li et al. (2020) or alternative transport mechanisms such as systemic redistribution or endocytosis. These observations highlight the need for further research to clarify the uptake pathways and behaviour of NPs across different crop species under environmentally realistic conditions.

## Conclusion

This study is the first focusing on the behaviour, uptake and impact of environmentally realistic MNPs (size, concentration, type and surface characteristics) on crop plants (wheat and tomato) using both individual and mixed exposure conditions, providing ecologically relevant insight at the soil-crop interface. We demonstrated that plants significantly reduce the mobility (and leaching) of MPs in soil through root interaction, suggesting their role in retaining MPs in upper layers of the soil. Our results also showed that smaller MPs are more mobile in soil and are therefore more likely to leach into surrounding environments (e.g. groundwater). Overall, PET fibres at concentrations reported in biosolids had more significant impacts on plant growth and chlorophyll content, particularly in tomato plants. This confirms species-specific impacts of MPs on plants; however, more research is required to investigate the mechanisms of these impacts. The combined exposure of MNPs at concentrations typical in biosolids led to significant reduction of root and shoot length in tomato plants, highlighting the importance of mixture exposure and toxicity for evaluating the environmental risk of MNPs to plants. Uptake of aged NPs was observed into root and lower stem tissue of tomato and wheat as well as tomato leaf. This raises concern about the accumulation of smaller-sized plastics in edible plant tissues, posing possible risks to food safety and human health. Future studies should investigate the full trajectory of different types of environmental NPs from root uptake to fruit tissues and evaluate any potential physiological or biochemical effects at both the cellular and whole-plant level. Our findings also highlight the importance of considering realistic exposure scenarios in risk assessment of MNPs and calls for urgent attention to the environmental implication of increasing MNP contamination in agricultural soils. Effective management strategies are needed to control and minimise MNP input into agricultural ecosystems and to safeguard crop quality and human health.

## Supplementary Information

Below is the link to the electronic supplementary material.

ESM 1 (DOCX 5.22 MB)

## Data Availability

Some of the data generated in this study are provided in the Supporting Information. Additional data are available from the corresponding author upon reasonable request.
