# Supplementary material for: Microplastic uptake and impacts on crops under realistic exposure: implications for soil–plant systems
Source: Environ Sci Pollut Res Int. 2026 Apr 1;33(13):6259–73. doi: 10.1007/s11356-026-37686-z (PMC13095943; doi:10.1007/s11356-026-37686-z)
Supplement: Supplementary file 1 — (DOCX 5.22 MB) [file 11356_2026_37686_MOESM1_ESM.docx]

**Microplastic uptake and impacts on crops under realistic exposure: implications for soil–plant systems**

Shima Ziajahromi*, Chris Pratt, Nikol Slynkova, Frederic D.L. Leusch

Australian Rivers Institute, School of Environment and Science, Griffith University, Gold Coast Campus, Southport, Gold Coast, Qld 4222, Australia

Table of Contents

[Section S1 3](#_Toc204603316)

[Table S1 3](#_Toc204603317)

[Table S2 3](#_Toc204603318)

[Section S2 4](#_Toc204603319)

[Section S3 5](#_Toc204603320)

[Figure S2 6](#_Toc204603321)

[Table S4 6](#_Toc204603322)

[Figure S3. 7](#_Toc204603323)

[Figure S4. 8](#_Toc204603324)

[Figure S5 8](#_Toc204603325)

[Figure S6 9](#_Toc204603326)

[Figure S7 10](#_Toc204603327)

[Figure S8 11](#_Toc204603328)

**Section S1. MNP particle size analysis:**

The size of the micro- and nanoplastic particles was determined using a laser diffraction spectroscopy (Malvern Mastersizer 3000, Malvern Instruments), which detects particles between 0.01 to 2000 µm. To achieve an appropriate obscuration value for each polymer type, a sufficient quantity of particles was introduced into the dispersion unit containing ultrapure water. To reduce measurement errors, the particles were initially dispersed in a vial with 50 mL of ultrapure water and 50 µL of Tween 20 (a 0.1% v/v; Sigma Aldrich, Germany) solution. The prepared suspensions were then sonicated for 30 minutes before measurement. The results obtained from the Mastersizer 3000 are available in the supplementary data section (Table S1).

**Table S1**. Size distribution of MNPs.

| **MP type** | **Nominal size range (µm)** | **Actual Size distribution (µm)** |
| --- | --- | --- |
| PET fibres | 25-500 | 37.8 – 824 |
| PE fragment | 25-500 | 28.1-552 |
| PS bead | ∼0.4 | 0.37-0.41 |

**Table S2**. Soil type analysis (analysed by Malvern Mastersizer 3000)

| **Fraction** | **%** |
| --- | --- |
| Clay (<2um) | 14.07 |
| Silt (2 - 50um) | 65.44 |
| Very fine sand (50-100um) | 7.1 |
| Fine sand (100-250um) | 4.98 |
| Medium sand (250-500um) | 5.54 |
| Coarse sand (500-1000um) | 2.86 |
| Very coarse sand (1000-2000um) | 0.02 |
| Total sand (50-2000um) | 20.5 |

**Table S3**. Average concentration of MPs measured in MP-spiked soils

| **MP type** | **Nominal concentration / g** | **Actual concentration/ 50 g** | **Actual concentration/ g** | **Range** |
| --- | --- | --- | --- | --- |
| PE fragments | Low: 0.2 μg | Low: 8.21 μg | Low: 0.16 μg | -20% |
|  | High: 9.6 mg | High: 386.6 mg | High: 7.75 mg | -19.1 |
| PET fibres | Low: 0.6 μg | Low: 36.5 μg | Low: 0.71 μg | +18.3. |
|  | High: 0.9 mg | High: 60.75 mg | High: 1.1 mg | +21.6% |

**Section S2. Microplastic UV Aging process:**

Accelerated aging of microplastics fibers and fragments as well as nanoplastics beads was conducted by exposing the particles to UVB radiation under laboratory condition in an aging chamber designed and developed in-house (Griffith University Electronic Workshop) (Figure S1). The aging chamber was placed in a fume hood to safely exhaust any ozone that may be generated. The prepared MPs were put in glass petri dishes and placed in the chamber equipped with ultraviolet lamps (UVB 312 nm) with 97 W/m^2^ irradiance for 380h (16 d) as recommended by a previous study (Wang et al. 2020). To ensure that all particles were aged uniformly, particles were physically mixed every 24 h. The oxidation level was determined based on the oxidation of level of MPs (through analysis of parameters such as carbonyl index using FTIR) found in biosolid and agricultural soil samples during our previous works (Ziajahromi et al. 2024a, Ziajahromi et al. 2024b).

**
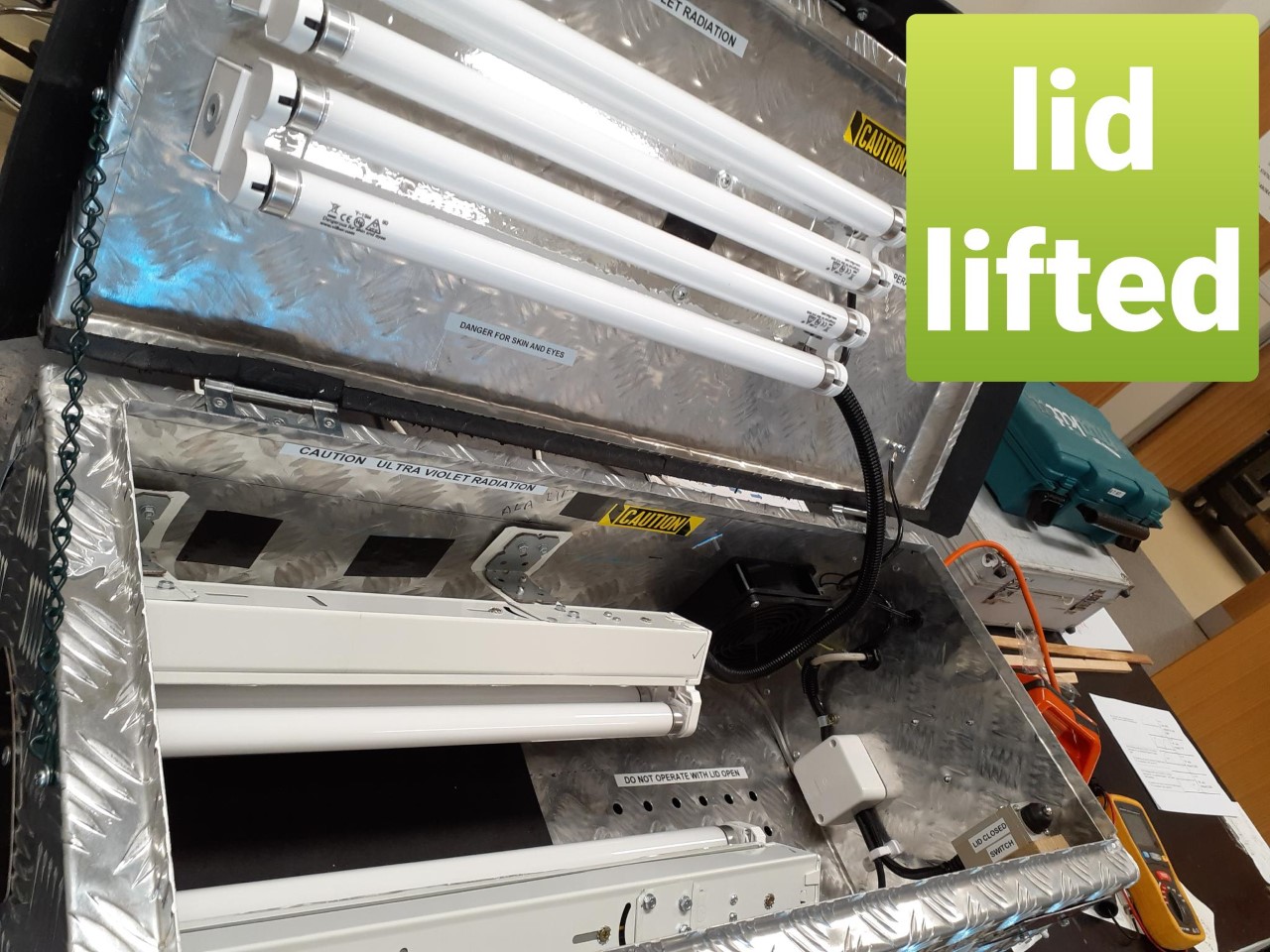
Figure S1**. Aging chamber.

**Section S3: Measuring the actual concentration of MPs in MP-spiked soils:**

To confirm the actual concentration of MPs in the MP-spiked soils. MPs were spiked at the desired concentration with 500 g of dried soil in 500 mL glass jars according to the method describe in Section 2.2. From each jar 50 g of soil samples were processed. This was done with two replicates. The MP-spiked soils were thoroughly mixed before taking a sub-sample. The samples were then digested using an oxidative method already optimized and validated in our previous studies Briefly, 10 mL of ultrapure water was added to each beaker and mixed using a magnetic stirrer until the samples were mixed well, followed by adding 20 mL of 30% (v/v) hydrogen peroxide (H_2_O_2_, Chem Supply, Australia) and 20 mL of 0.05 M FeSO_4_. The digestion was continued by adding 30 mL of H_2_O_2_ (in 10 mL increments).

Following the completion of oxidative digestion, the samples underwent density separation and filtration process to extract MPs for further analysis. Sodium iodide solution with an initial density of 1.8 g/cm^3^ (Chem Supply, Australia) was added to each tube, mixed well manually, and then centrifuged for 5 min. The buoyant particles were filtered through 25 and stainless steel filters, and the filters were dried in the oven at 50 °C.


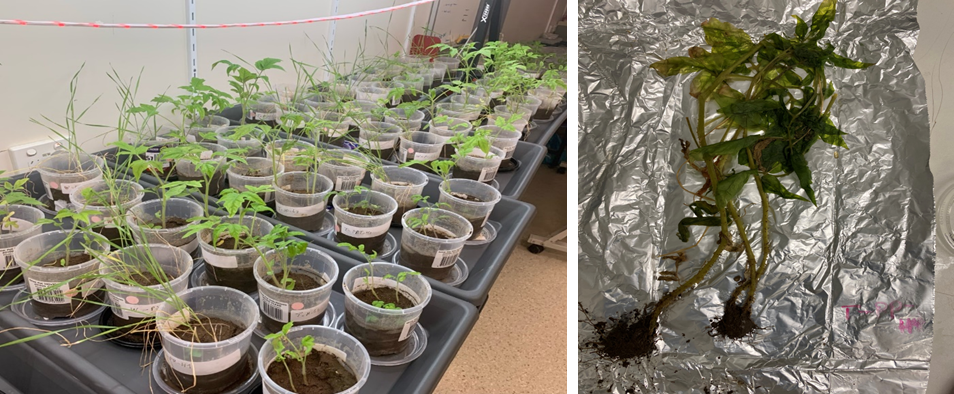


**Figure S2**. Plant experimental set up (left side) and the collected plant at the end of the experiment (right side).

**Table S4**. concentration of fertilizers added to each pot.

| **Element** | **g per pot** | **Salt** | **% target element in salt** | **mg salt per pot** |
| --- | --- | --- | --- | --- |
| N | 0.0318 | Urea | 47 | 67.65957447 |
| P | 0.01272 | DAP | 20 | 63.6 |
| K | 0.01908 | K2SO4 | 45 | 42.4 |

**
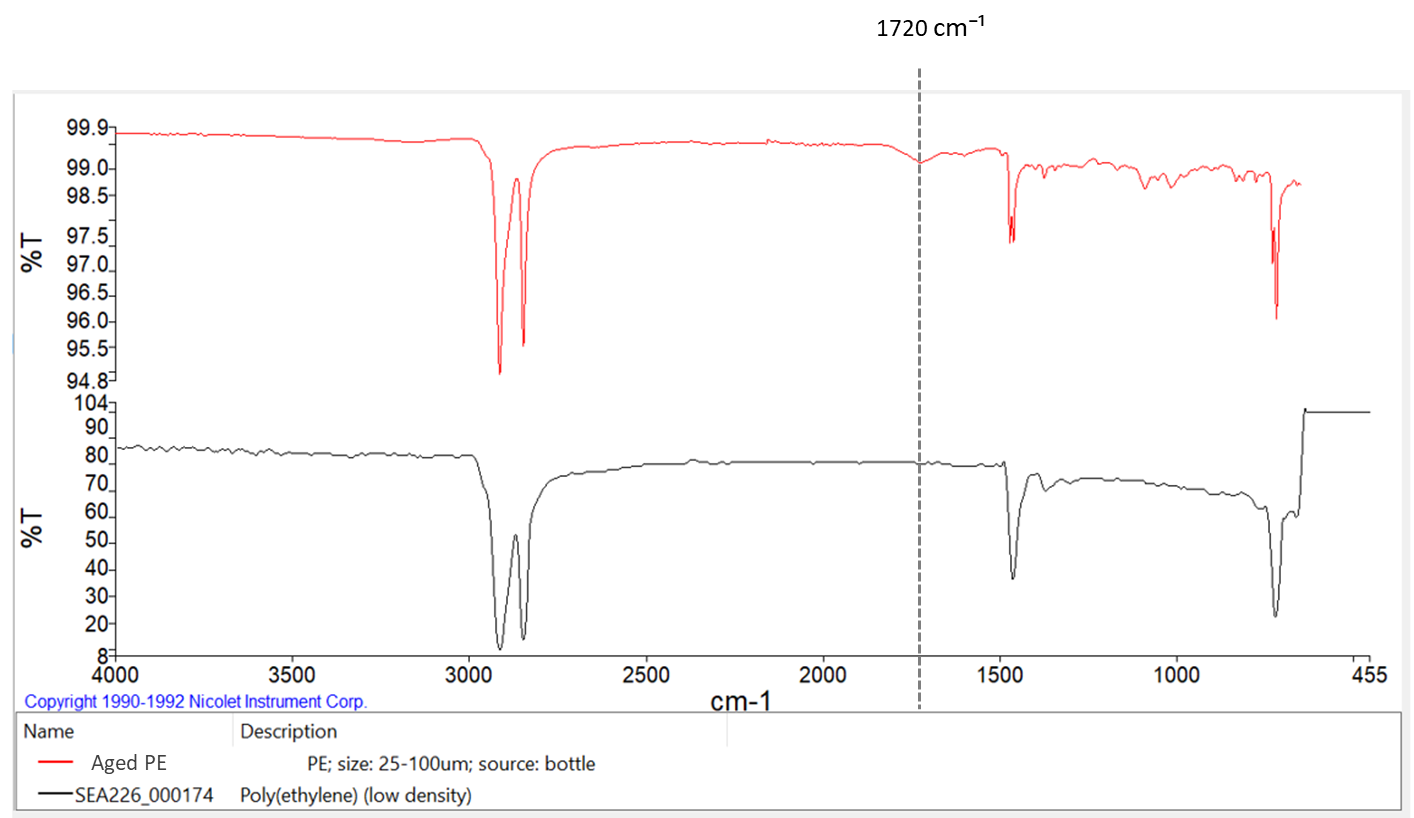
**

**
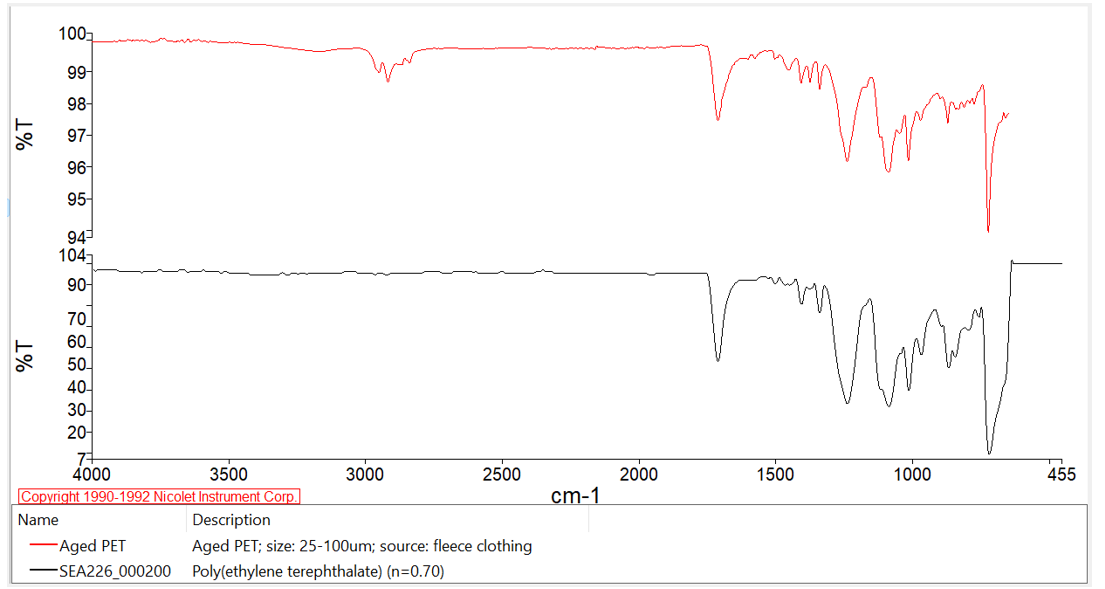
**

**Figure S3**. Zeta potential and FTIR spectra of aged and pristine MNPs.


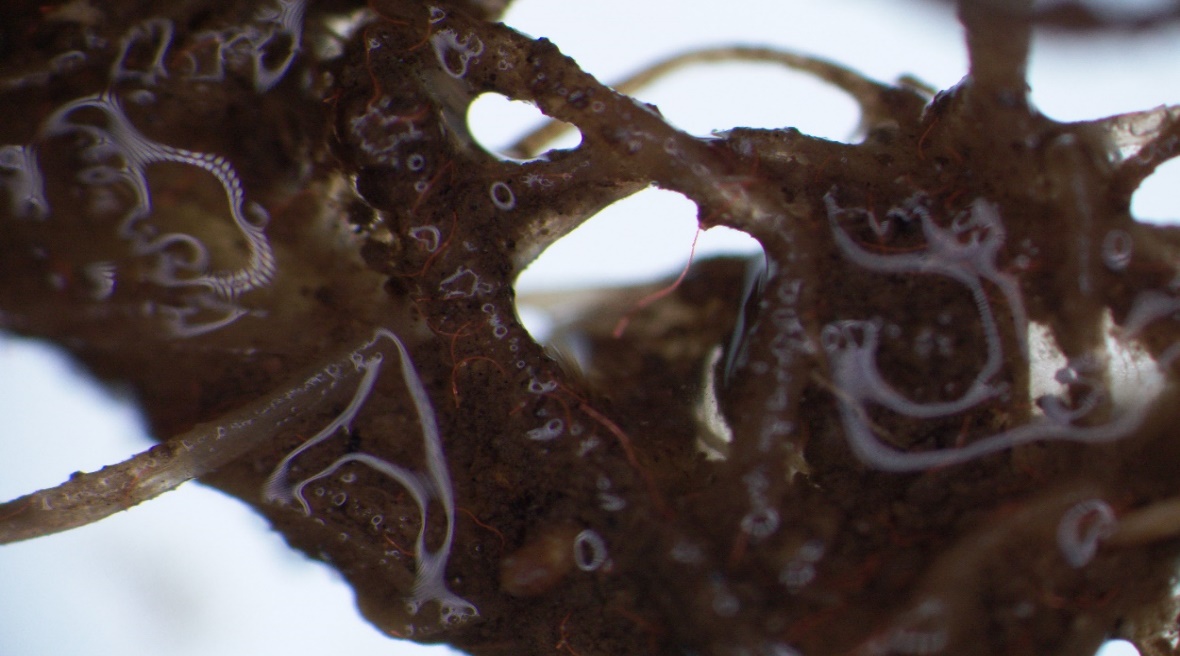


**Figure S4**. MP PET fibres entangled around tomato toot.

**
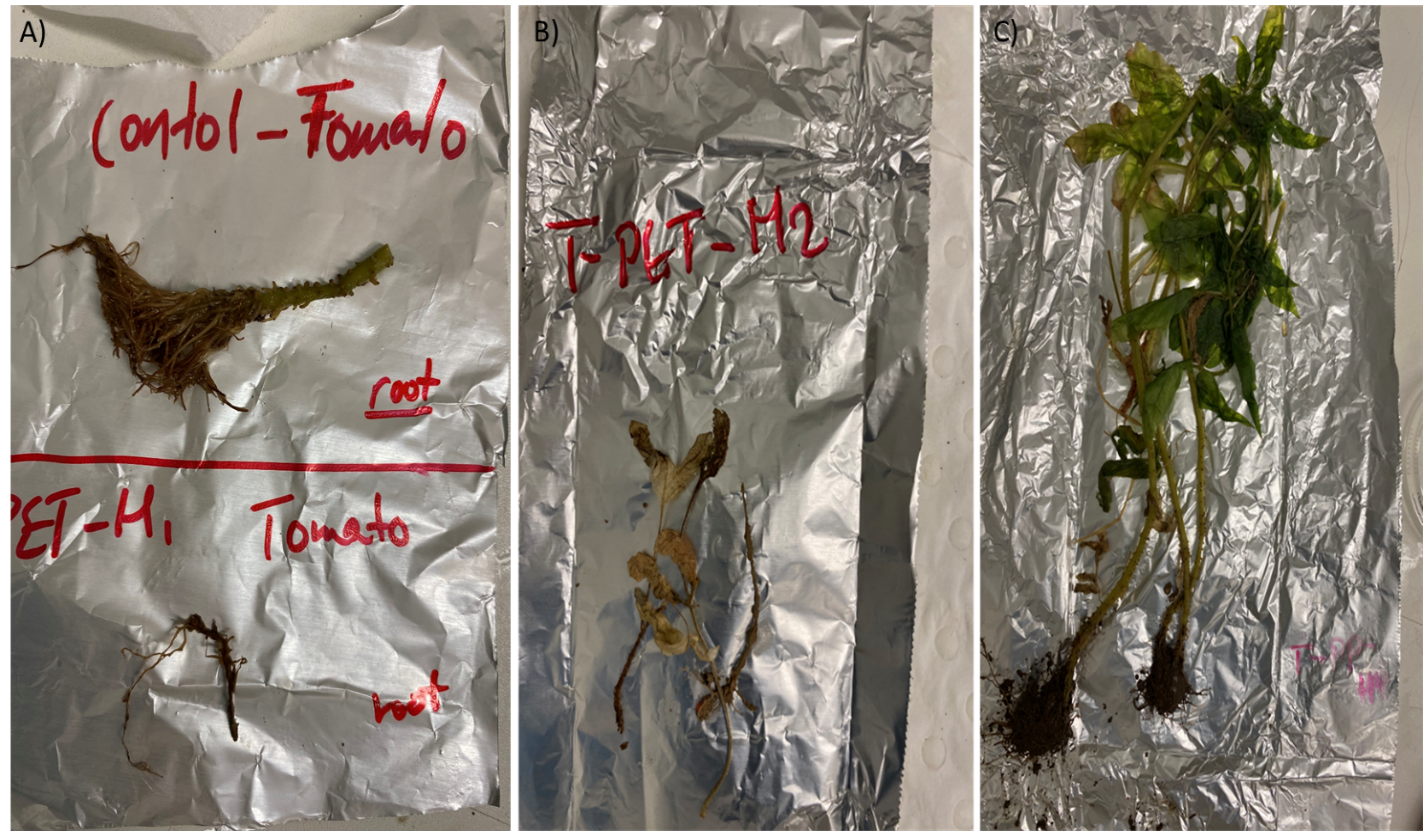
**

**Figure S5**. Phenotypic images of tomato plant exposed to PET fibres (high concentration). A) Root of exposed plant (bottom) compared to the root of control plant (top). B) above the ground part of totmato exposed to PET, C) above the ground part of tomato of control treatment.


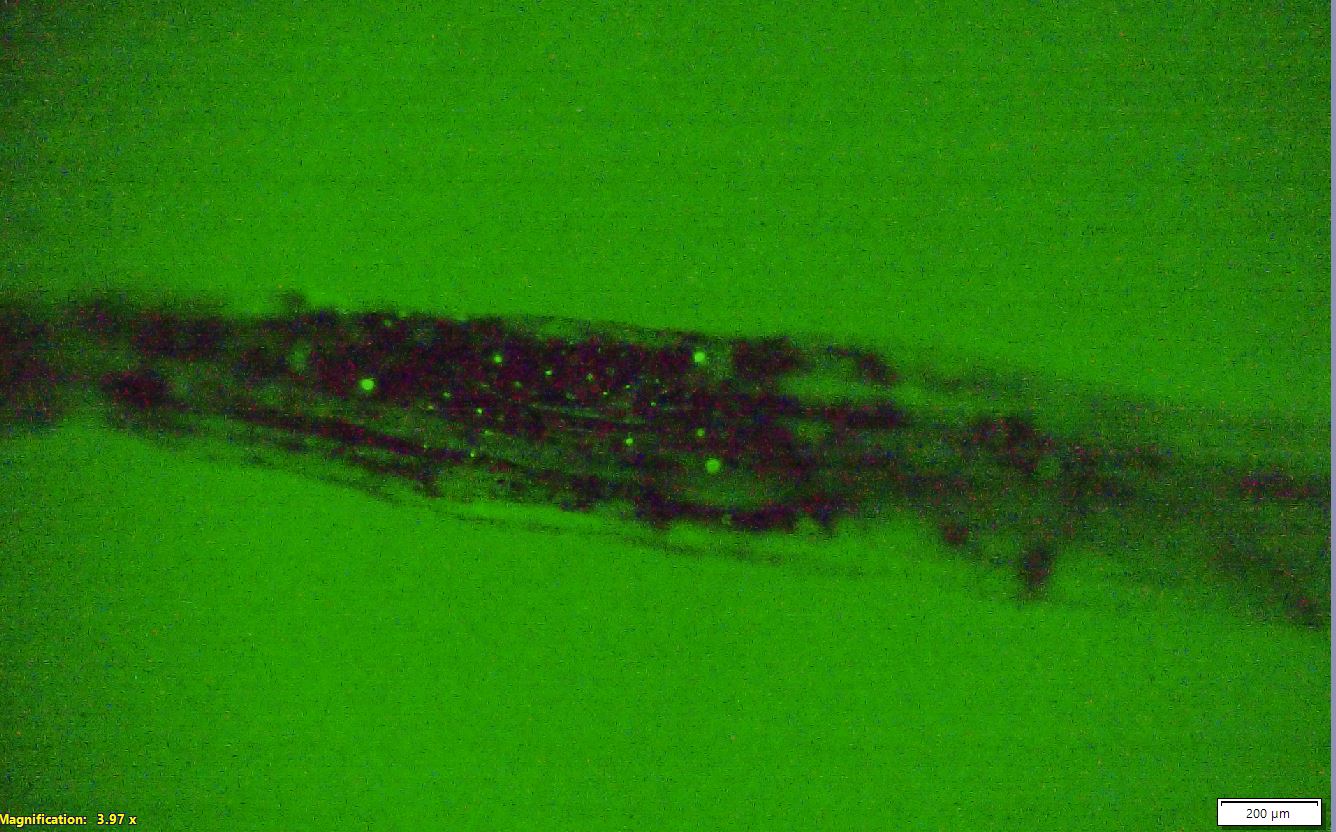

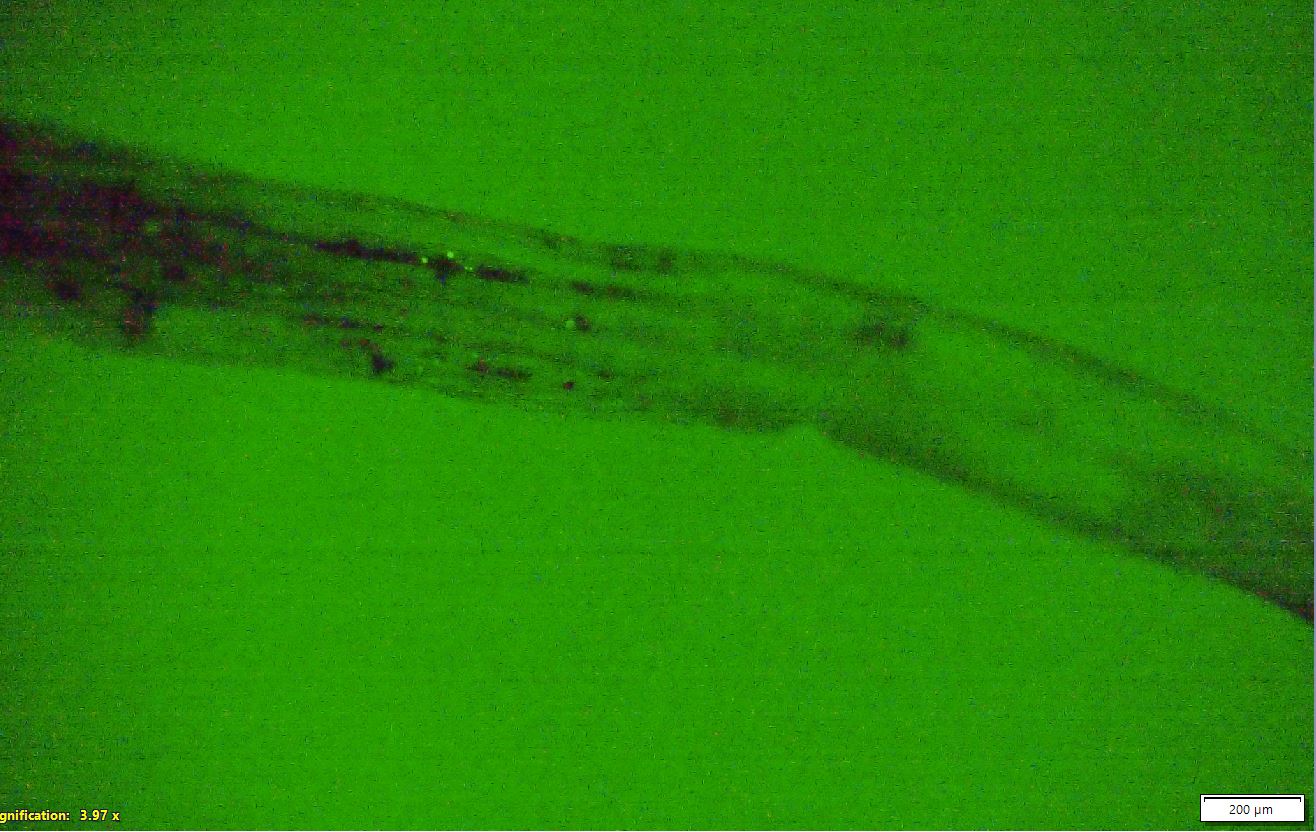


**B)**

**A)**


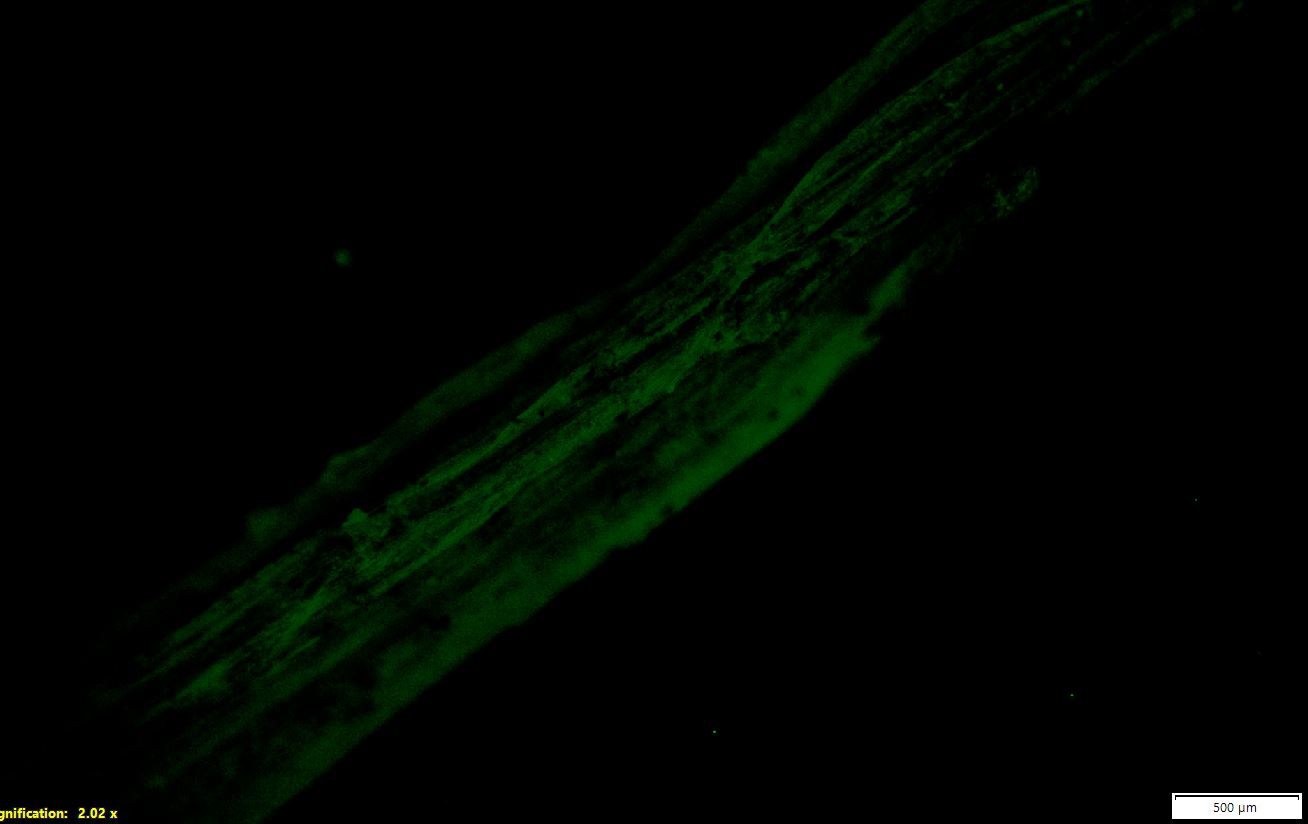


**C)**

**Figure S6. A)** and **B)** Accumulation of PS NPs at 0.05 μg/g in the stem base region of wheat plant. **C)** Control treatment. Red arrow refers to the NP accumulated in multiple parts of the tissue.

**
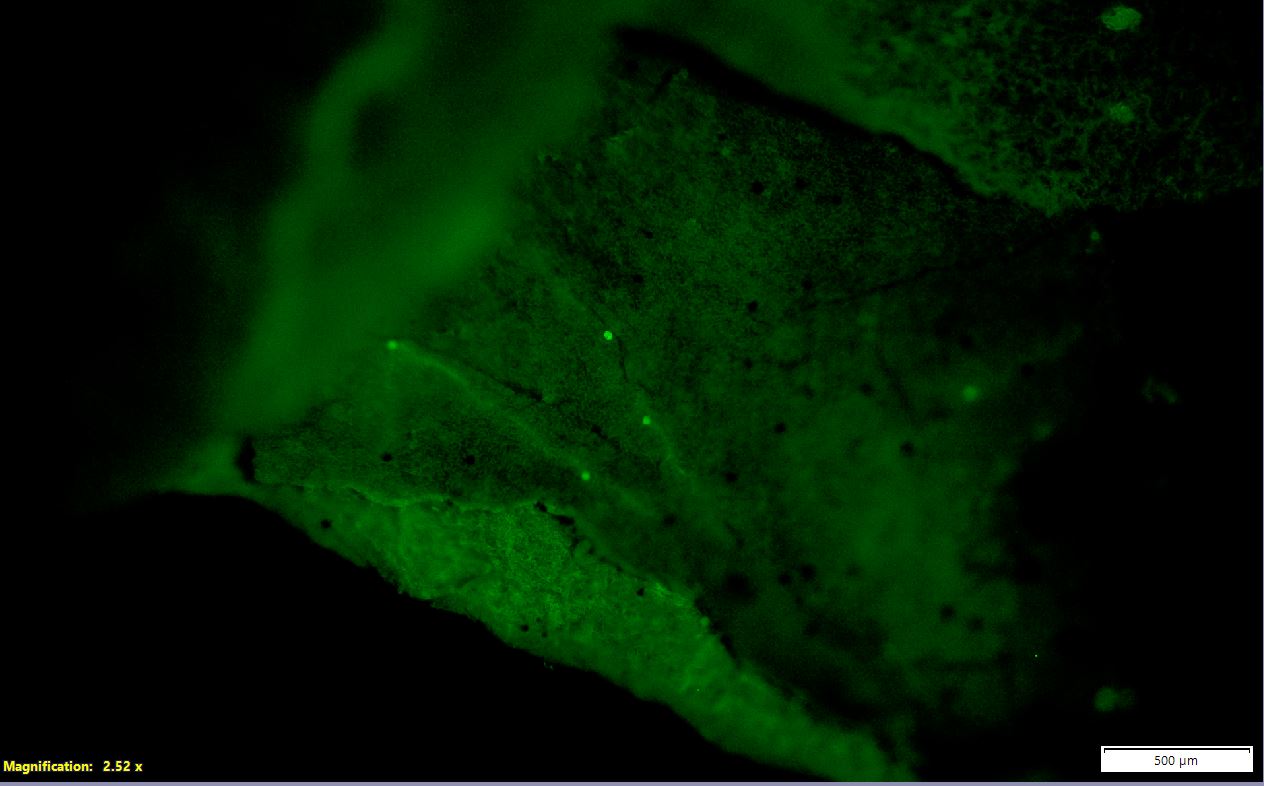
**

**
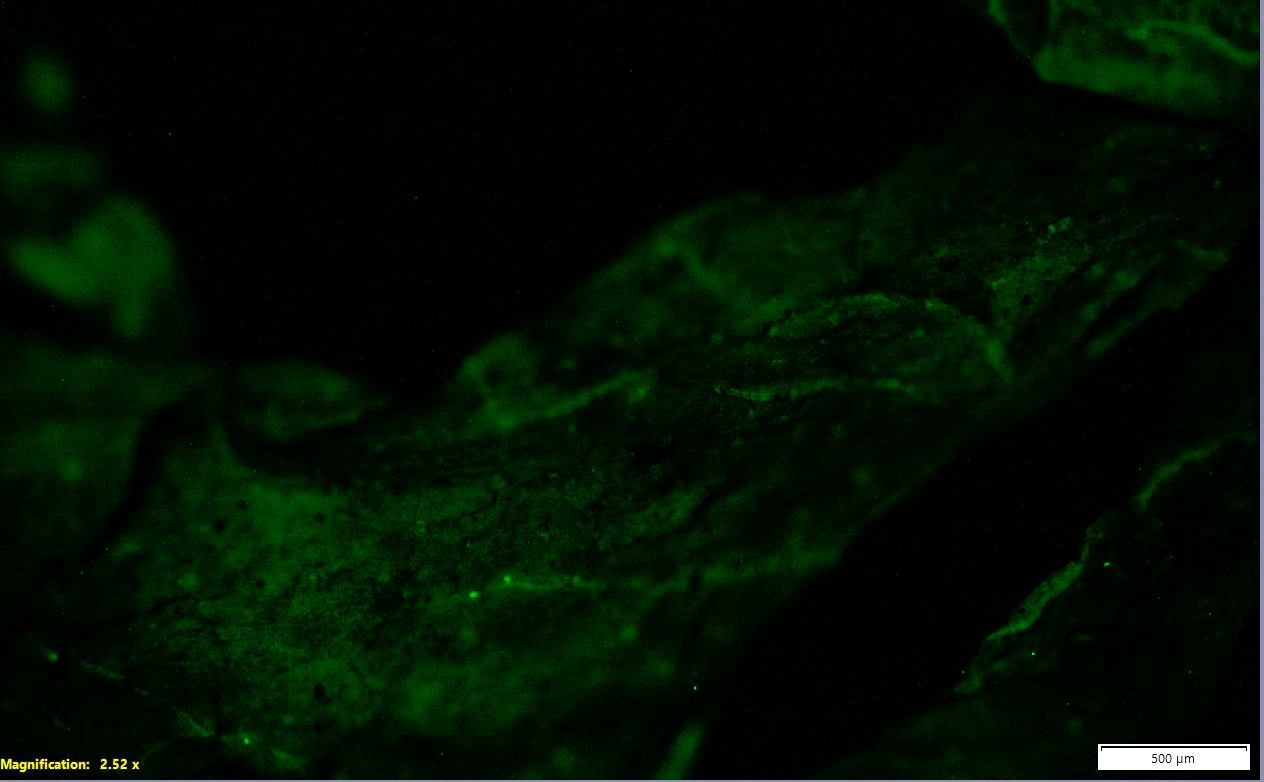
**

**Figure S7.** Small spots of PS NP at 0.05 μg/g observed in tomato leaf tissue. Red arrows refer to the NP accumulated in multiple parts of the tissue.

**
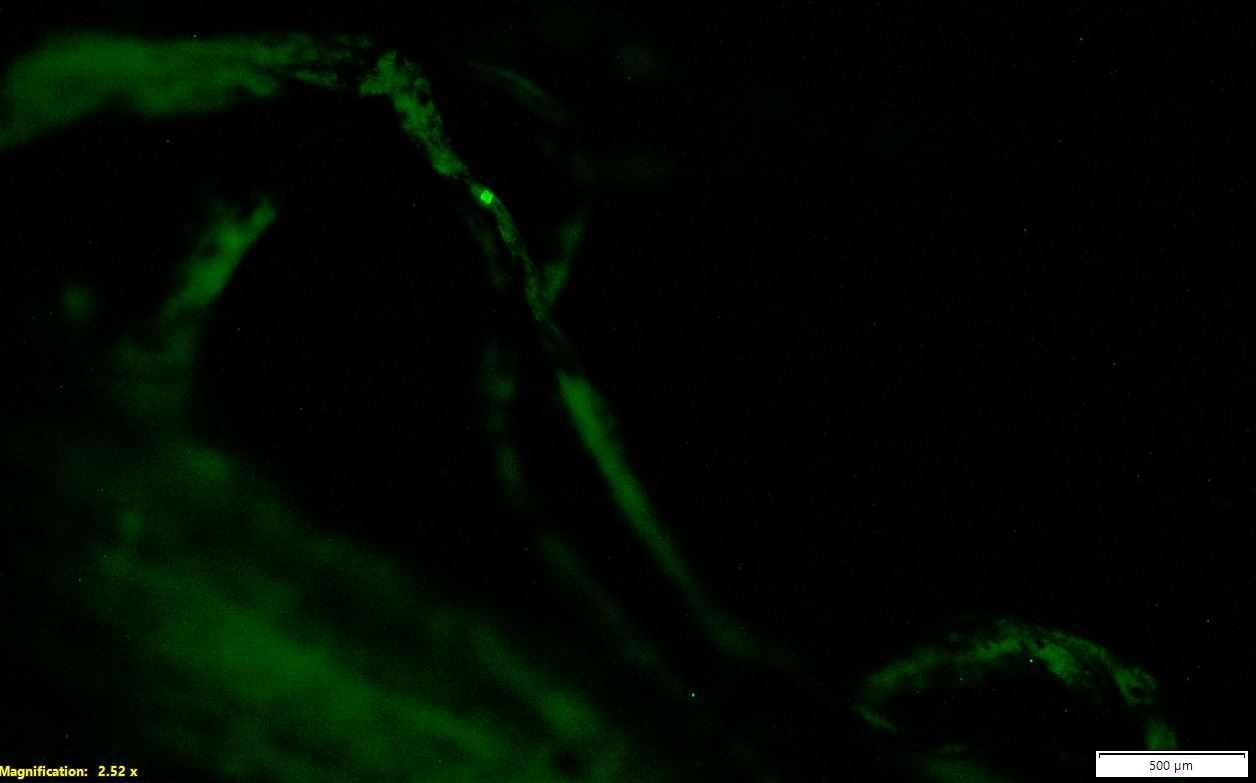
**

**A)**


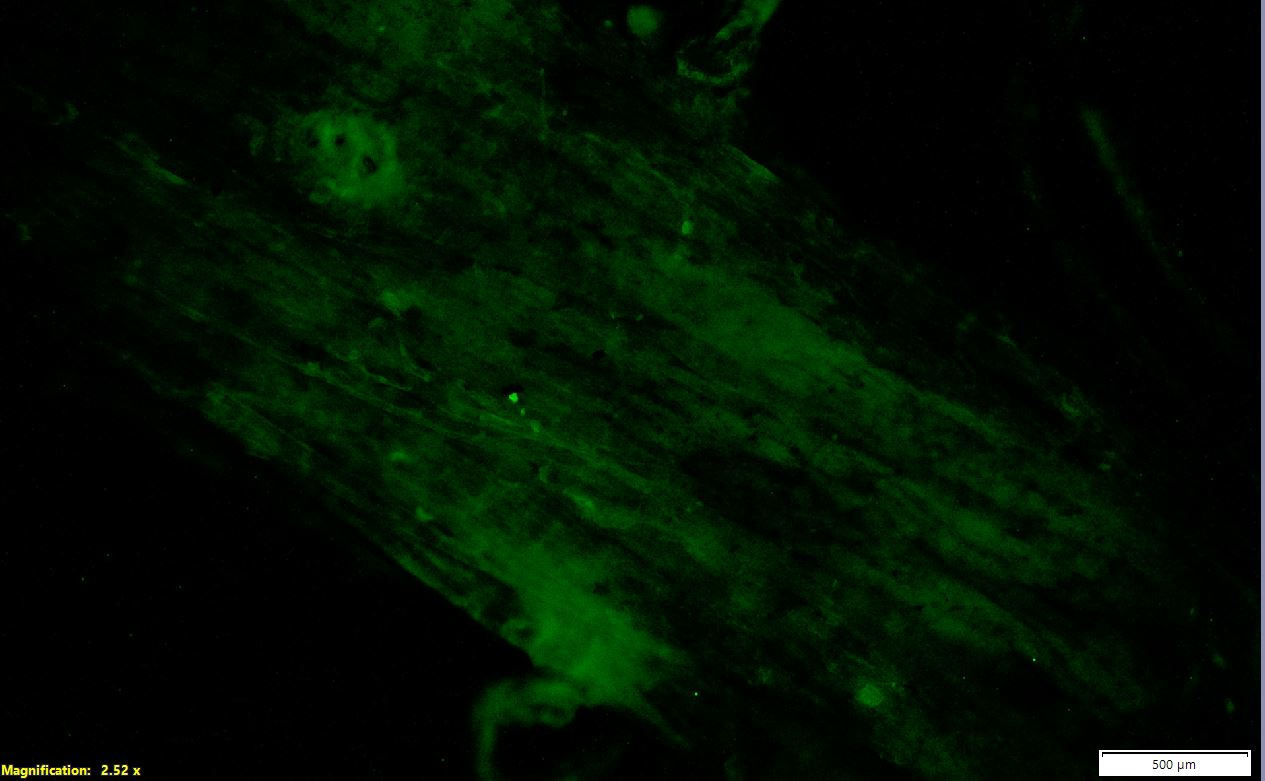


**B)**


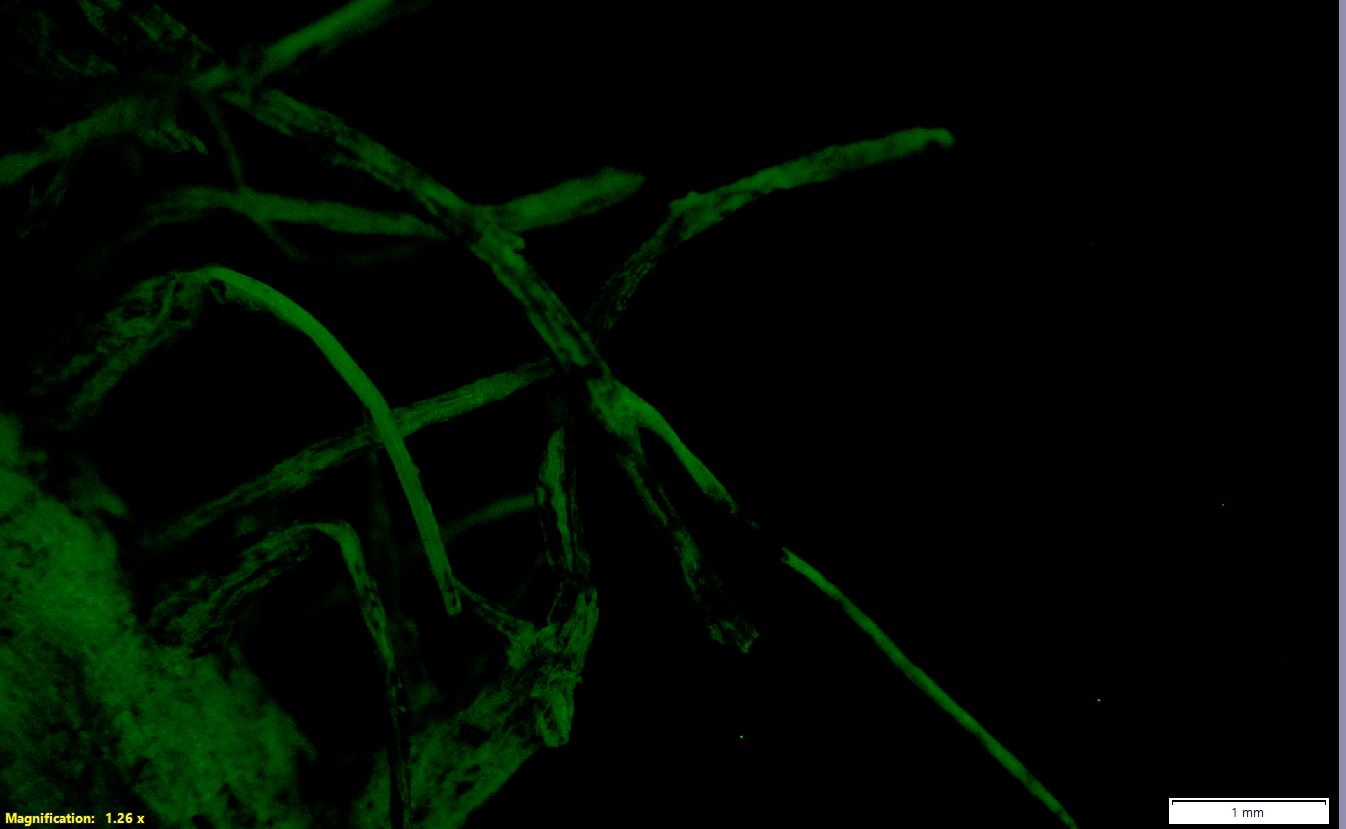


**C)**

**
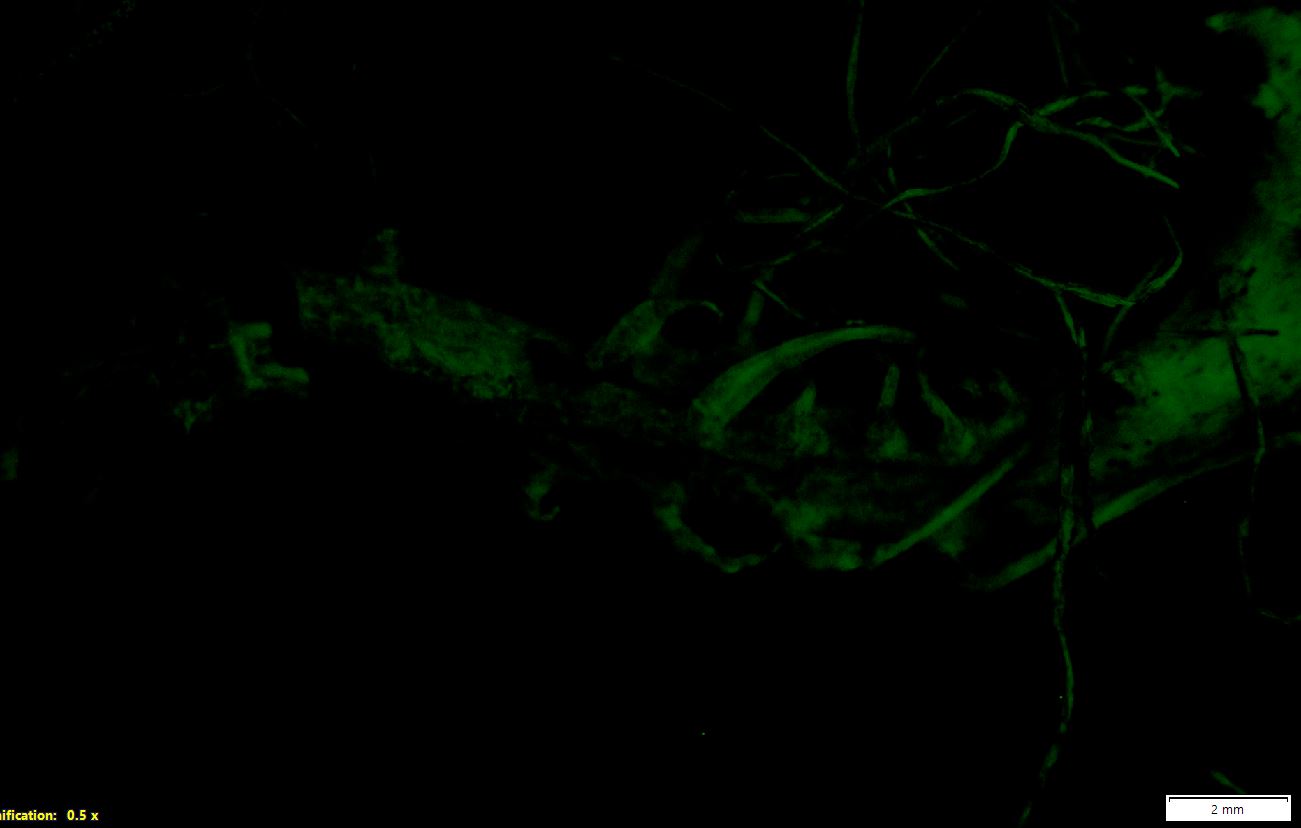
**

**D)**

**Figure S8. A)** and **B)** PS NP at 0.05 μg/g observed in tomato root tissue. **C)** Control treatment- root hair, **D)** Control treatment- root area. Red arrows refer to the NP in multiple parts of the tissue.

**References**

Wang, Q., Zhang, Y., Wangjin, X., Wang, Y., Meng, G. and Chen, Y. (2020) The adsorption behavior of metals in aqueous solution by microplastics effected by UV radiation. Journal of Environmental Sciences 87, 272-280.

Ziajahromi, S., Lu, H.C., Dwyer, J., Fernandes, M., Griffith, M. and Leusch, F.D. (2024a) Transport and Accumulation of Microplastics from Biosolids to Australian Agricultural Soils: Detection of Microplastics Down to 1 mum. Environ Sci Technol.

Ziajahromi, S., Slynkova, N., Dwyer, J., Griffith, M., Fernandes, M., Jaeger, J.E. and Leusch, F.D.L. (2024b) Comprehensive assessment of microplastics in Australian biosolids: Abundance, seasonal variation and potential transport to agroecosystems. Water Res 250, 121071.
